# Acute pancreatitis associated with duodenal obstruction induced by groove pancreatitis: A case report

**DOI:** 10.1097/MD.0000000000026139

**Published:** 2021-06-04

**Authors:** Jiayan Li, Qianyi Liu, Zhishang Liu, Chuan Cen, Yuyu Yang, Jianming Ye, Li Xu, Xiji Lu, Dongfeng Chen, Weishan Ruan

**Affiliations:** Department of Gastroenterology, Zhongshan People's Hospital, Zhongshan City, China.

**Keywords:** acute pancreatitis, case report, duodenal obstruction, groove pancreatitis

## Abstract

**Rationale:**

Groove pancreatitis (GP) is a rare form of chronic pancreatitis. Since GP presents with nonspecific symptoms, it can be challenging to diagnose. Duodenal obstruction is often caused by malignant diseases; however, when associated with acute pancreatitis, it is rarely induced by groove pancreatitis.

**Patient's concerns:**

A 56-year-old man who presented with acute pancreatitis complained of recurrent upper abdominal discomfort. His concomitant symptoms included abdominal pain, postprandial nausea, and vomiting. Contrast-enhanced computed tomography (CT) of the abdomen showed thickening of the duodenum wall. Gastrointestinal radiographs and upper gastrointestinal endoscopy showed an obstruction of the descending duodenum.

**Diagnosis:**

The pathologic diagnosis was groove pancreatitis.

**Interventions:**

The patient underwent gastrojejunostomy to relieve the obstruction.

**Outcomes:**

The patient had an uneventful recovery with no complications.

**Lessons:**

Groove pancreatitis should be considered in the differential diagnosis of patients presenting with acute pancreatitis and duodenal obstruction. These data can help to make a precise diagnosis and develop an appropriate treatment plan.

## 1 Introduction

Groove pancreatitis is an uncommon form of chronic pancreatitis that affects the groove region between the pancreatic head, duodenum, and common bile duct. It often presents in middle-aged males with a history of alcohol abuse.^[1]^ Pathogenesis is still unclear and clinical presentation is not specific. It is a rare disease, and most of the cases tend to be diagnosed after surgery. As a consequence, the diagnosis of GP can be challenging. Imaging features such as sheet-like hypodensity in the pancreaticoduodenal groove, medial duodenal wall thickening, and cystic changes in the duodenal wall are important to suggest the diagnosis of groove pancreatitis.^[2]^ GP can be cured by conservative medical treatment, and surgery is reserved only for patients with persistent and severe clinical symptoms or for a definitive exclusion of malignancy.^[3]^ The differential diagnosis of GP includes duodenal cancer, pancreatic carcinoma, cholangiocarcinoma, or acute pancreatitis with phlegmon along the groove area. In the present case, acute pancreatitis with duodenal obstruction caused by groove pancreatitis could be easily misdiagnosed.

## 2 Case presentation

A 56-year old male was admitted to the hospital with acute epigastric abdominal pain. He was diagnosed with “acute pancreatitis,” for which he received conservative treatment in the duration of one week. Three days after his discharge, he presented to our clinic with recurrent sharp abdominal pain accompanied by nausea and vomiting, which worsened following the food intake. He had a history of hypertension. The patient denied tobacco or alcohol use. Family history was not significant.

Physical examination revealed epigastric tenderness. Laboratory tests showed alanine aminotransferase 64 U/L (normal range 9–50 U/L), and gamma-glutamyl transferase 93 U/L (normal range 10–60 U/L). His serum amylase and lipase levels, aspartate aminotransferase, serum carbohydrate antigen (CA) 19-9, and carcinoembryonic antigen (CEA) levels were within normal limits.

**Figure 1:**
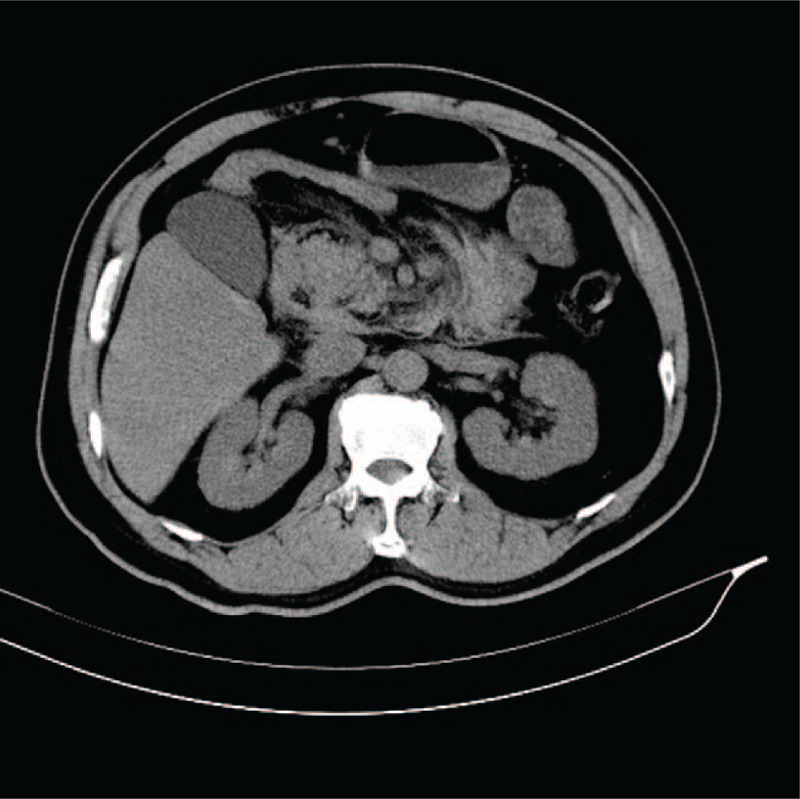
Computed tomography scan showing enlargement of the pancreatic head, as well as infiltraion of peripancreatic fat.

**Figure 2:**
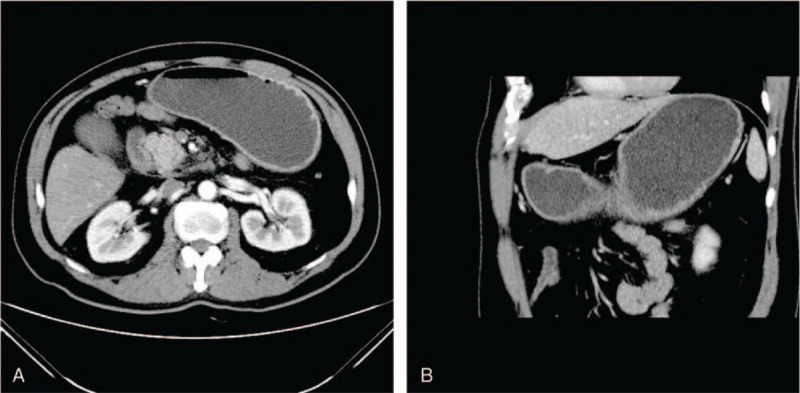
(A) Contrast-enhanced computed tomography showing improvement in swelling of the pancreatic head, Thickening of the duodenal wall. (B) Gastric outlet obstruction.

**Figure 3:**
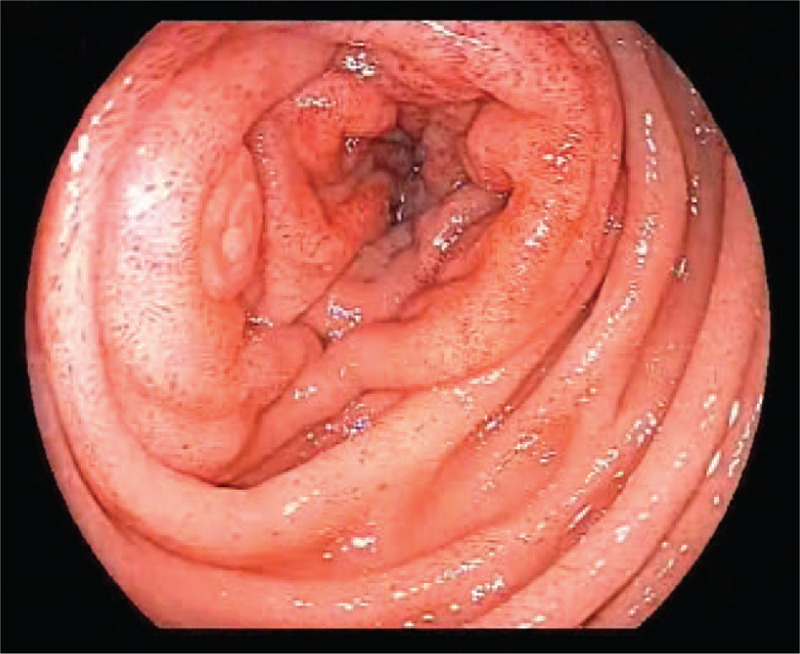
Upper gastrointestinal endoscopy showing that the second duodenal portion was edematous and narrow.

**Figure 4:**
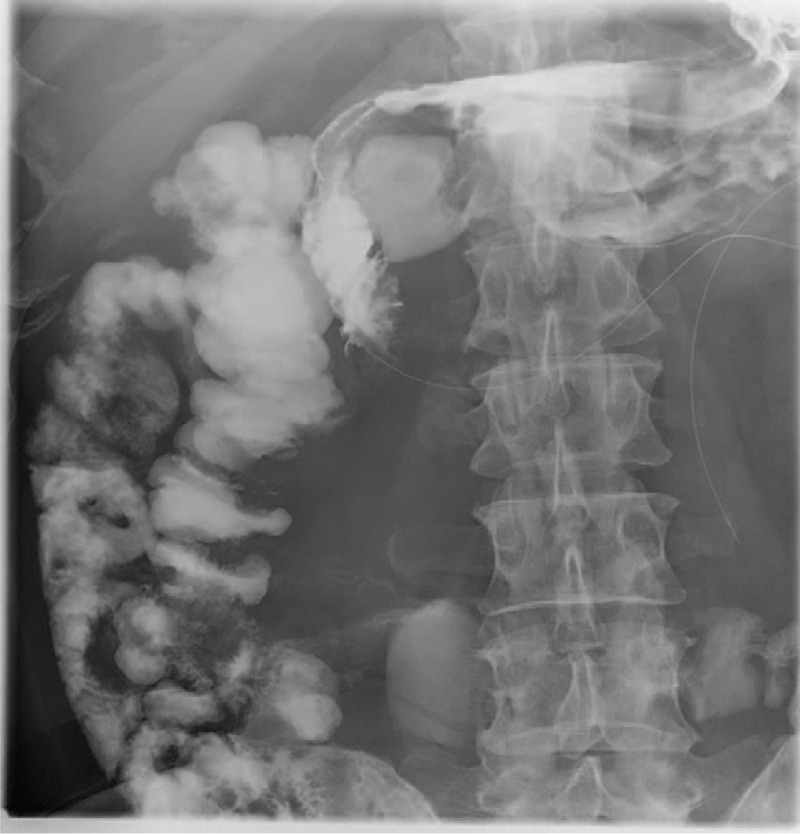
Gastrointestinal radiographs showed that the descending duodenum was narrow.

**Figure 5:**
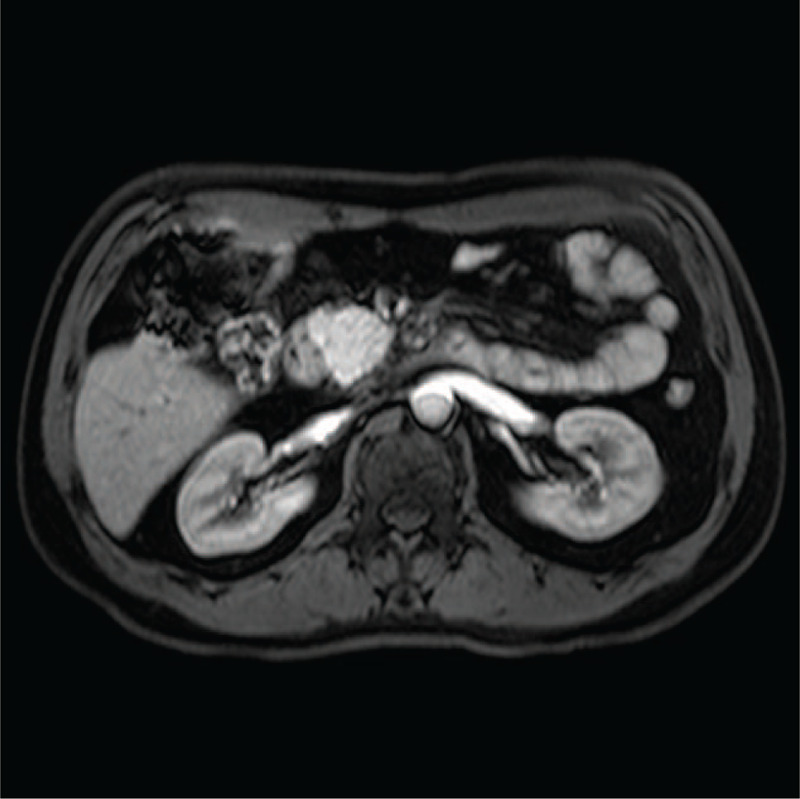
Contrast-enhanced magnetic resonance imaging showing thickening and luminal narrowing of the descending part of the duodenum.

During his first hospitalization, the CT scan demonstrated enlargement of the pancreatic head, as well as infiltraion of peripancreatic fat, which indicated acute pancreatitis (Fig. 1). During his second hospitalization, contrast-enhanced computed tomography showed improved swelling of the pancreatic head, gastric outlet obstruction, and thickening of the duodenal wall (Fig. 2). An upper gastrointestinal endoscopy was performed, revealing a scar formed by a duodenal bulbar ulcer. Furthermore, the second duodenal portion was edematous and narrow (Fig. 3), whereas histology was negative for malignancy. Gastrointestinal radiographs showed that the descending duodenum was narrow (Fig. 4). Contrast-enhanced magnetic resonance imaging (MRI) and magnetic resonance cholangiopancreatography (MRCP) showed thickening and luminal narrowing of the descending part of the duodenum without any obvious mass in the pancreas or duodenum (Fig. 5). The biliary system was normal. The pancreatic duct was not dilated.

**Figure 6:**
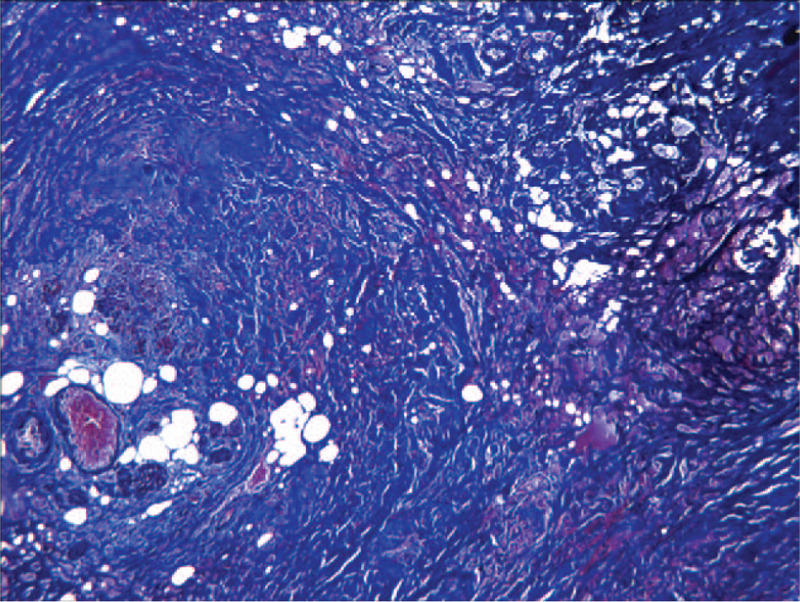
Histopathologic examination showing collagen fiber hyperplasia with Masson trichrome stain in the groove area (×10).

**Figure 7:**
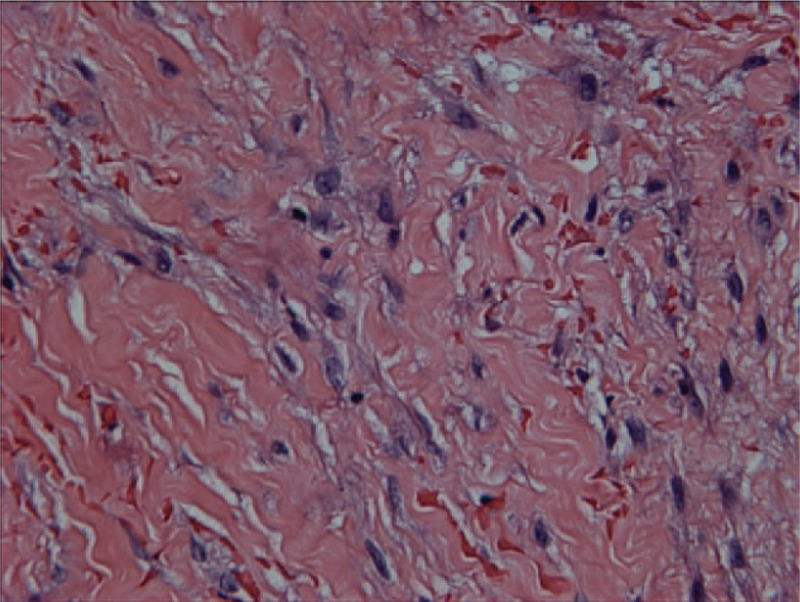
Histopathologic examination showing myofibroblastic proliferation in the groove area (×60).

Since it is not possible to exclude pancreas or duodenal carcinoma, surgery is used for diagnosis as well as for treatment. Gastrojejunostomy was performed to relieve the obstruction. During the surgery, extensive scarring and widening of the pancreaticoduodenal groove were found, whereas no mass or enlarged lymph nodes were seen in the pancreas or duodenum. The pathology of the groove area was negative for malignancy. The histopathologic examination revealed collagen fiber hyperplasia with Masson trichrome stain (Fig. 6) and myofibroblastic proliferation in the groove area (Fig. 7), which was compatible with groove pancreatitis.

Postoperative recovery was uneventful. Our patient recovered well, having no symptoms after 3 years of follow-up.

## 3 Discussion

Groove pancreatitis was first reported by Becker in 1973.^[4]^ GP is a distinct subset of chronic pancreatitis, which involves the duodenal wall in the vicinity of the minor duodenal papilla, the adjacent pancreatic parenchyma, and the potential space (groove) between them.^[5]^ Becker and Mischke further classified groove pancreatitis into two forms: pure, which involves the groove area only, with preservation of the pancreatic parenchyma and main pancreatic duct and segmental, which involves both the groove and the head of the pancreas with stenosis of the pancreatic duct causing upstream dilatation.^[6]^ These forms accounted for 8.9% and 15.5%, respectively, of 123 pancreaticoduodenectomies performed in patients with chronic pancreatitis.^[7]^ The pathogenesis of groove pancreatitis remains unclear, but anatomical or functional obstruction of the minor papilla is one of the considered issues. Several factors, such as chronic alcohol abuse, smoking, peptic ulcers, gastric resection, true duodenal-wall cysts, and pancreatic heterotopia in the duodenal wall, have been identified as potentially related to this condition.^[7,8]^ One proposed theory about the etiology of GP is that it is due to either the primary or secondary obstruction of the accessory duct of Santorini and the minor papilla. Indeed, cystic dilatation of the accessory duct (“Santorinicele”) is a frequent finding at pathology.^[9]^ Heterotopic pancreatic tissue as well as Brunner's gland hyperplasia that infiltrates the wall of the second duodenum may lead to partial obstruction of the minor papilla as the duct of Santorini. Finally, other inflammatory processes involving the proximal duodenum such as ulcerations from peptic ulcer disease have also been implicated as a cause of GP.^[10]^ Our patient had a duodenal bulbar ulcer with no history of alcohol abuse.

The clinical manifestations of groove pancreatitis include upper abdominal pain, weight loss, postprandial vomiting, and nausea due to duodenal stenosis, while jaundice rarely occurs.^[11]^ Serum pancreatic and hepatic enzyme levels are sometimes slightly elevated. Tumor markers, such as CEA and carbohydrate antigen (CA) 19-9, are usually within normal limits.^[5]^

Thickening and scarring of the duodenal wall close to the minor papilla usually cause stenosis of the second portion of the duodenum. Cystic changes in the thickened duodenal wall are distinctive, and typically contain clear fluid, necrotic and granular material, or stones.^[12]^ Becker and Mischke have found dduodenal-wall cysts in 49% of patients with groove pancreatitis.^[6]^ The key histologic criteria include dilated ducts and pseudocystic changes in the duodenal wall; duodenal submucosal fibrosis extending to the adjacent soft tissue in the groove area and pancreas; variable Brunner gland hyperplasia forming a thick layer with surrounding smooth muscle and myofibroblastic proliferation.^[13]^ At histology the most common finding in GP is duodenal Brunner's gland hyperplasia of the duodenal mucosa which contributes to the thickening of the duodenal wall. Microscopic examination reveals that heterotopic pancreatic tissue occurs in both the submucosa or muscular is propria of the duodenal wall.^[14]^

Characteristic imaging findings on CT include reactive parietal thickening along the medial aspect of the descending duodenum. The thickened duodenal wall often shows prominent enhancement and, depending upon the degree of duodenal thickening/scarring and consequential luminal compromise, upstream gastric dilatation may be found.^[8]^ Additional findings include cystic-like lesions in the duodenal wall or in the groove area. A sheet-like mass between the head of the pancreas and the duodenum associated with duodenal wall thickening is the most characteristic finding on magnetic resonance imaging.^[13]^ When the duodenum of patients with groove pancreatitis is too narrow to perform gastrointestinal fiberscopy, magnetic resonance cholangiopancreatography is a useful diagnostic option.^[11]^

Endoscopic ultrasound (EUS) is also effective in diagnosing groove pancreatitis. Yet, EUS makes insertion impossible due to duodenal stenosis, and the accuracy of EUS is dependent on operator and experience. The potentialities of the EUS are multiple, as it can also detect thickening and stenosis of the second duodenal part with intramural cysts, smooth stenosis of the common bile duct; and in the segmental form, heterogeneous hypoechoic mass, enlargement of the pancreatic head, with calcifications or pseudocyst and dilatation of the main pancreatic duct.^[14]^ EUS-guided fine-needle aspiration is a valuable approach for excluding pancreatic cancer.

If the diagnosis of GP is clear, patients may be conservatively, endoscopically, or surgically treated. Conservative treatment options include stop smoking/alcohol consumption, recovery of pancreatic function, and analgesics. Yet, such treatments tend to be only temporarily effective.^[15]^ Relapsing episodes of pancreatitis may not be prevented in cases with an anatomic disturbance of pancreatic juice outflow.^[16]^

Endoscopic management includes cyst fenestration or drainage, and pancreatic ductal stenting has been reported to produce symptomatic relief.^[17,18]^

Surgery is the treatment of choice for when symptoms do not improve, or when the condition is too difficult to be distinguished from pancreatic carcinoma. Pancreaticoduodenectomy is the usual initial surgical approach for the treatment of these patients, whereas some authors recommend performing a preliminary gastroenterostomy to relieve symptoms of obstruction.^[19]^ Pancreatoduodenectomy still has considerable mortality (2%–3%) and morbidity rates (37%–40%).^[20]^ Lekkerkerker et al^[21]^ have reported 50% (4/8) adverse events in patients with GP after pancreaticoduodenectomy, where 13% (1/8) died postoperatively because of hypovolemic shock. A gastroenteroanastomosis may be a solution for patients with marked duodenal stenosis without intractable pain or in patients who are unfit for pancreatectomy.^[1]^ This less complex surgical approach allows pancreatic tissue to be spared and reduces surgical morbidity. Another surgical approach used for GP is a pancreas-preserving duodenectomy.^[22]^ Although this enables the preservation of pancreatic tissue, long-term resolution of symptoms is less than with pancreaticoduodenectomy.^[22]^

Groove pancreatitis presenting as acute pancreatitis associated with duodenal obstruction has not been previously reported in the literature. Groove pancreatitiscan be challenging to differentiate using clinical features, imaging, and laboratory markers. Therefore, for some cases, surgery might be eventually required. If patients present with acute pancreatitis when a duodenal obstruction is present, groove pancreatitis should be considered. In order to facilitate the correct diagnosis and appropriate management, MRI, CT scan, EUS, and even biopsy should be considered. The treatment of choice is conservative, although a surgical intervention can sometimes be required.

## Author contributions

WR contributed to design, and critically revised manuscript. JL contributed to acquisition and analysis of data, drafted manuscript, and selected items. QL participated in conception, and critically revised manuscript. ZL helped to acquisition of data, and selected items. CC analyzed data, and drafted manuscript. YY contributed to conception, and critically revised manuscript. JY analyzed data, and critically revised manuscript. LX helped to acquisition of data, critically revised manuscript. XL participated in interpretation of data, and selected items. DC contributed to interpretation of data, critically revised manuscript. All authors read and approved the final manuscript.

**Conceptualization:** Qianyi Liu, Yuyu Yang, Weishan Ruan.

**Data curation:** Jiayan Li, Zhishang Liu, Li Xu, Xiji Lu.

**Formal analysis:** Jiayan Li, Zhishang Liu, Chuan Cen, Jianming Ye, Xiji Lu, Dongfeng Chen.

**Writing – original draft:** Jiayan Li, Chuan Cen.

**Writing – review & editing:** Qianyi Liu, Yuyu Yang, Jianming Ye, Li Xu, Dongfeng Chen, Weishan Ruan.
